# Genotyping of *Chlamydophila psittaci* in Human Samples

**DOI:** 10.3201/eid1212.051633

**Published:** 2006-12

**Authors:** Edou R. Heddema, Erik J. van Hannen, Birgitta Duim, Christina M.J.E. Vandenbroucke-Grauls, Yvonne Pannekoek

**Affiliations:** * University of Amsterdam, Amsterdam, the Netherlands;; † Saint Antonius Hospital, Nieuwegein, the Netherlands;; ‡ VU University Medical Center, Amsterdam, the Netherlands

**Keywords:** *Chlamydophila psittaci*, ornithosis, bacterial outer membrane proteins, molecular sequence data, genotype, birds, dispatch

## Abstract

*Chlamydophila* (formerly *Chlamydia*) *psittaci* genotypes A, B, C, and a new genotype most similar to the 6BC type strain were found in 10 humans with psittacosis by outer membrane protein A gene sequencing. Genotypes B (n = 3) and C (n = 1) are endemic in nonpsittacine European birds. These birds may represent an important part of the zoonotic reservoir.

Psittacosis is a zoonosis caused by infection with Chlamydophila (formerly Chlamydia) psittaci, an obligate intracellular bacterium. C. psittaci is divided into 8 serovars (A–F, M56, and WC) and at least 9 genotypes. Sequence analysis of the outer membrane protein A (ompA) gene is the most accurate method for identifying all known genotypes (*1*). All genotypes are associated with specific bird groups from which they are predominantly isolated (*2**,*3). The prevalence of different genotypes of C. psittaci that cause infection in humans is unknown. In this study, we genotyped all C. psittaci PCR-positive human samples available in our laboratory.

## The Study

Ten human samples positive for C. psittaci DNA in our previously described real-time PCR assay were characterized by ompA gene sequencing (*4*). These samples were collected from 2002 through 2005 and included 4 sputum, 4 bronchoalveolar lavage, 1 throat swab, and 1 serum. All samples were obtained from symptomatic psittacosis patients admitted to hospitals in the Netherlands. All patients had pneumonia, and 6 required treatment in an intensive care unit. The DNA of 1 outbreak strain, which infected as many as 10 people, was tested only once in this study. One of the samples was obtained from a patient who has been previously described (*5*).

DNA purification was performed by using the guanidinium thiocyanate-silica–extraction procedure (*6*). Genotyping was performed essentially as previously described (*7*). Briefly, a part of the ompA gene was amplified with primers CPsittGenoFor (5´-GCT ACG GGT TCC GCT CT-3´) and CPsittGenoRev (5´-TTT GTT GAT YTG AAT CGA AGC-3´). These primers are located in the conserved regions of the ompA gene that contains the 4 variable domains (VDs). On the basis of published ompA sequence of the C. psittaci 6BC type strain (GenBank accession no. X56980), the resulting amplicon should have a size of 1,041 bp. PCR products were analyzed by agarose gel electrophoresis, and the expected 1,041-bp amplicon was excised from the gel. DNA was extracted from the gel and reamplified for 20 cycles, and amplicons were analyzed for size by agarose gel electrophoresis. C. psittaci ORNI (genotype A) strain and a C. abortus strain were used as positive controls. Calf thymus DNA was used as a negative control.

If the ompA gene could not be amplified with this procedure, a nested PCR with primers CPsittFinner (5´-CGC TCT CTC CTT ACA AGC C-3´) and CPsittRinner (5´-GAT CTG AAT CGA AGC AAT TTG-3´) was used. The amplified product (n = 8) or the nested PCR product (n = 2) were subjected to sequence analysis (BigDye Terminator, version 1.1, cycle sequencing kit, Applied Biosystems, Foster City, CA, USA). Overlapping sequences were obtained with 6 sequencing primers: CPsittGenoFor and CPsittGenoRev, inner primers CPsittFinner and CPsittRinner, and primers CPsittHFor (5´-TCT TGG AGC GTR GGT GC-3´) and CPsittHRev (5´-GCA CCY ACG CTC CAA GA-3´).

The resulting sequences were aligned, and a similarity index based on the translation of the 984-bp gene fragment was calculated. Similarity (1 – distance) was calculated by using the pairwise distance method generated by MEGA3 (*8*). Reference ompA genotype sequences A–F and the ompA sequence of the C. psittaci 6BC type strain available in GenBank (accession nos. AY762608–AY762612, X56980, and AF269261) were included in this analysis (*1**,**9*).

All 10 isolates could be genotyped. The ompA sequence of 5 isolates was identical to the sequence of reference genotype A, 3 isolates were identical to genotype B, and the ompA sequence of 1 isolate was identical to genotype C. One isolate had a novel ompA sequence type that was 99.4% similar to the genotype A reference but more similar to the C. psittaci 6BC type strain (99.7%). Two nonsynonymous mutations were present in this sequence compared with reference genotype A. A substitution of thymine for adenine in VD 1 resulted in Ser instead of Thr at amino acid position 92 of the ompA amino acid sequence, which is identical to that found in genotype C. A substitution of cytosine for guanine, also located in VD 1, resulted in Gln instead of Glu at amino acid position 117, as found in genotype B and strain 6BC (numbering according to the ompA amino acid sequence of the C. psittaci 6BC strain, GenBank accession no. X56980). We designated this new variant C. psittaci 05/02 and deposited the sequence in GenBank (accession no DQ324426). Two genotype B strains, 3 genotype A strains, and the novel genotype 05/02 strain were obtained from patients admitted to an intensive care unit.

## Conclusions

To our knowledge, ours is the first report of a series of genotyped C. psittaci strains isolated from symptomatic, hospitalized patients. These 10 samples reflect approximately one third of all cases reported each year in the Netherlands (*10*). From the genotypes that we identified, we may infer the zoonotic reservoirs of C. psittaci in the Netherlands. The different genotypes of C. psittaci are associated, although not exclusively, with different birds from which they are mostly isolated. Genotype A is mainly found in psittacine birds and was the most prevalent genotype in our samples (*1**,**3*). C. psittaci 05/02 was most related to C. psittaci 6BC and the reference genotype A (strain VS1). Both reference strains have been classified as serovar A strains. On the basis of 2 restriction fragment length polymorphism patterns, Sayada et al. suggested that serovar A should be divided into 2 genogroups (*2*). Our isolate 05/02 is a new ompA gene sequence variant within this probably heterogeneous group.

Genotype B has been isolated mainly from feral pigeons and several other bird species; this genotype is considered endemic in European nonpsittacine birds (*3**,**11*). Genotype C has been isolated mainly from ducks, and we detected this genotype in 1 of our human samples. We did not find genotype D, which is prevalent among poultry, especially turkeys, or genotypes E and F. These last 2 genotypes are rare and found occasionally in birds (*1**,**11*). Imported psittacine birds, which carry mainly genotype A, have been proposed as the major source for human psittacosis infections (*12*). In our study, 4 of 10 isolates were genotypes B and C, which are rarely found in psittacine birds. This finding suggests that nonpsittacine birds may represent an underestimated source for human psittacosis cases.

We detected isolates of genotypes A, B, C, and a new genotype similar to the C. psittaci 6BC strain in a series of 10 C. psittaci–positive samples. Genotypes B and C are endemic in European nonpsittacine birds, which may represent an important part of the zoonotic reservoir for human psittacosis cases.
